# Decoupling Variable Capacitance and Diffusive Components of Active Solid–Liquid Interfaces with Flex Points

**DOI:** 10.1021/acsmeasuresciau.4c00057

**Published:** 2024-08-29

**Authors:** Liam Deehan, Ajeet Kumar Kaushik, Ganga Ram Chaudhary, Pagona Papakonstantinou, Nikhil Bhalla

**Affiliations:** † Nanotechnology and Integrated Bioengineering Centre (NIBEC), School of Engineering, Ulster University, 2-24 York Street, Belfast, Northern Ireland BT15 1AP, United Kingdom; ‡ Department of Environmental Engineering, Florida Polytechnic University, Lakeland, Florida 33805, United States; § Department of Chemistry and Centre of Advanced Studies in Chemistry, Panjab University, Chandigarh 160 014, India

**Keywords:** transducers, inflection points, cyclic voltammetry, biosensors, solid–liquid interfaces, electrode-capacitances

## Abstract

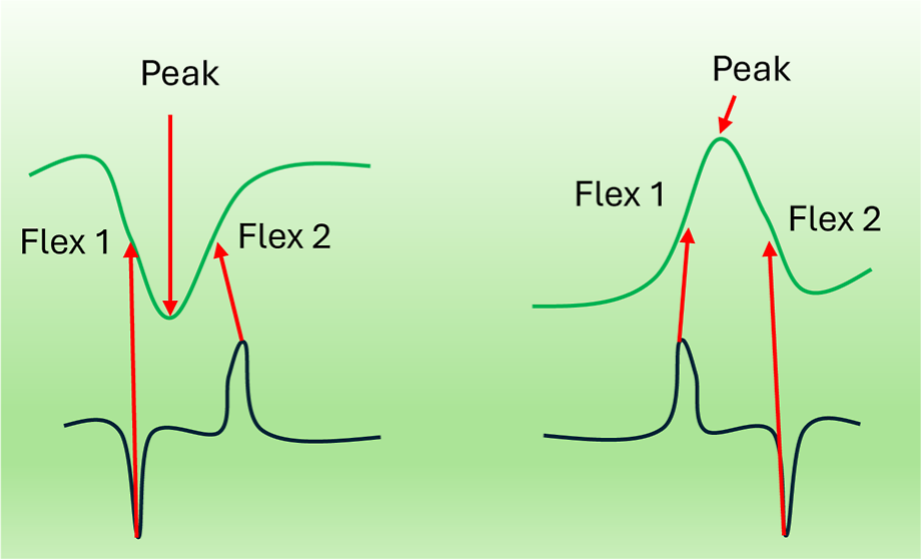

Understanding the current transport characteristics of electrode interfaces is essential for optimizing device performance across a wide range of applications including bio-/chemical sensing and energy storage sectors. Cyclic voltammetry (CV) is a popular method for studying interfacial properties, particularly those involving redox systems. However, it remains challenging to differentiate between electron movements that contribute to capacitive and diffusive behaviors. In this study, we introduce a technique called flex point analysis, which uses a single differentiation step to separate capacitive and diffusive electron movements at the electrode interface during a redox reaction. Our results show that the variable capacitance at the electrode surface exhibited both positive and negative values on the order of 10^–6^ (micro) Farad. This approach provides a clearer understanding of interfacial electron dynamics, enhancing the interpretation of CV data and potentially improving the design and optimization of related materials and devices.

## Introduction

Electrochemical techniques have long been integral to a wide range of applications from bio-/chemical sensing^1,2^ and materials design^3,4^ to energy storage devices^5,6^ and the study of fundamental interfacial properties.^7^ These methods provide essential insights into the behavior of chemical reactions at the electrode (solid) and solution (liquid) interfaces. Among the various amperometric and voltammetric electrochemical techniques,^8−11^ cyclic voltammetry (CV) stands out as one of the most powerful and versatile methods for investigating redox reactions and elucidating the underlying principles of electrochemical processes. In CV, a potential waveform with a repetitive shape is applied to an electrode immersed in an active solution.^1,10^ This potential undergoes a linear sweep over time to a predetermined limit, initiating oxidation and reduction reactions at the electrode interface. As the potential increases, analyte molecules undergo oxidation, losing electrons and generating a current.^12^ Upon reaching the switching potential, the reduction phase commences, during which the analyte gains electrons, leading to a reverse current.^13^ This generates a duck-shaped curve with distinct oxidation and reduction current peaks, allowing for the determination of crucial electrochemical parameters, such as peak potentials, peak currents, and scan rates, facilitating detailed investigations into various properties of the electrode interface.^10^

However, interpreting cyclic voltammograms (e.g., to understand electrode capacitance) presents significant challenges beyond identifying current peaks, primarily due to the complex task of distinguishing between diffusive and capacitive components within the characteristic duck-shaped curve. This complexity arises because capacitive and diffusive processes are often obscured by minute peak potential shifts^14−16^ and changes in the slopes of the oxidation and reduction currents.^17,18^ Traditional analysis methods, which predominantly focus on examining peaks in voltammograms, struggle to detect subtle changes in peak potentials or stand apart from the analysis of current slopes. As a result, existing methods limit our understanding of the underlying interfacial phenomenon, and in the case of the redox electrode system, this interface is referred to as the “active solid” (electrically active)–liquid interface.^19^

To address these challenges, this work develops an analytical framework aimed at enhancing the comprehension of redox peak dynamics, with a particular focus on decoupling the capacitive and diffusive currents associated with electrochemical processes or the electrically active solid–liquid interface. Essentially, our model identifies the flex points, where the curvature of the CV curve changes direction. This is achieved by locating the maximum value of the slope in the curve, which corresponds to the peak value of the first derivative in a given direction.^20,21^ These flex points allow us to effectively distinguish between the capacitive and diffusive components. These points also allow us to detect the variable capacitance at the interface of the electrode that varies with the rate of the reaction. In addition, the flex point analysis allows standardization of current slopes’ analysis. By standardizing the selection of voltage or current values using inflection points, our method significantly enhances the amount of information that can be extracted from CV measurements, surpassing the capabilities of simple peak analysis.^22^ This will allow for more accurate and meaningful comparisons of different CV curves, facilitating a deeper understanding of the electrochemical processes.^23,24^ As a result, researchers can better interpret the leading and trailing slopes of the duck-shaped CV curves, leading to improved insights and advancements in electrochemical studies and applications.

## Results and Discussion

**Figure 1:**
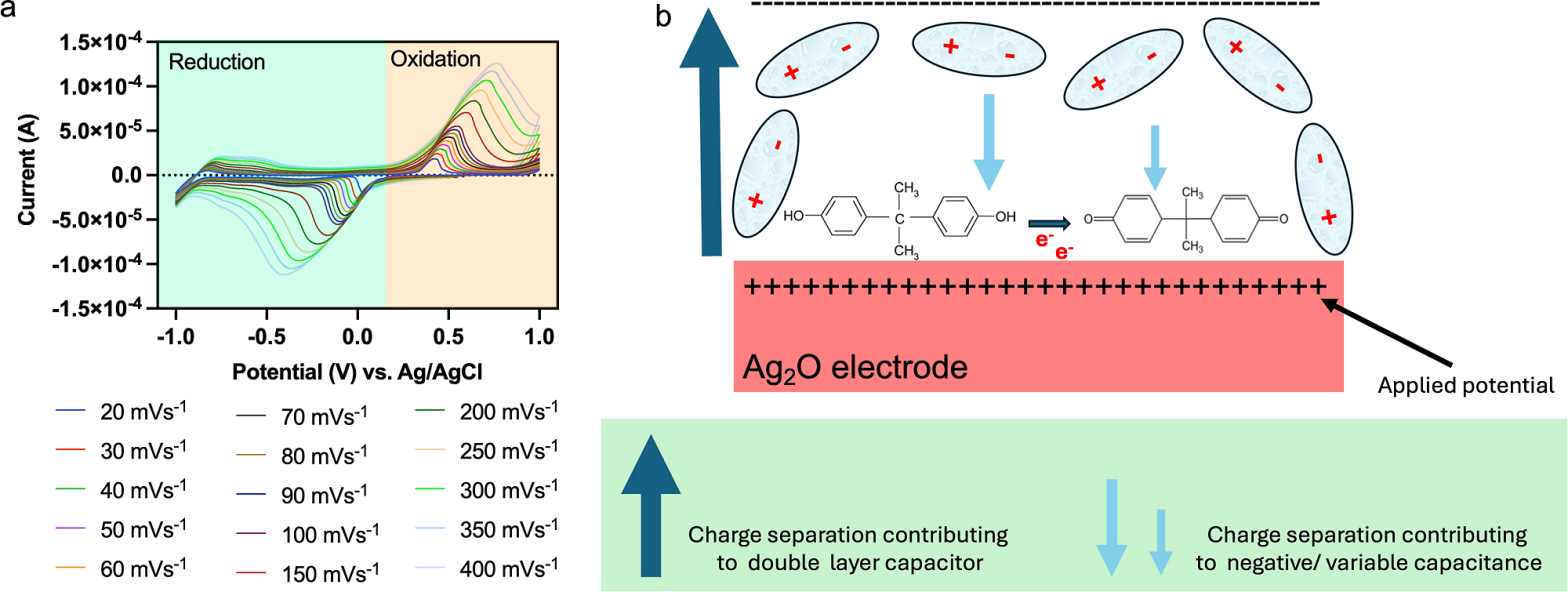
Experimental data: (a) cyclic voltammograms used for analysis in this work. The curves correspond to the oxidation and reduction of BPA on a platinum electrode decorated with Ag_2_O nanocubes. Note that this figure is reproduced with permission from ref (25) Copyright 2020 Elsevier. (b) shows generation of a charge by the redox reaction (the oxidation phase is shown in schematic), which enhances the electric field against the orientation of the dipoles forming the double-layer capacitance.

The CV experimental data were collected for various scan rates, ranging from 20 to 400 mV/s, based on our previous works by Chakraborty et al.^25^Figure 1a shows CV curves obtained by the oxidation and reduction of bisphenol A (BPA) on a platinum electrode decorated with Ag_2_O nanocubes, and Figure 1b is a schematic depicting the electrode surface state during the CV cycle. The concentration of BPA was 0.1 mmol of dm^–3^ BPA solution prepared in PBS (0.1 mol of dm^–3^ PBS, pH 7). The redox reaction on the electrode can be explained using eq 1
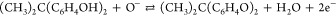
1

Here, (CH_3_)_2_C(C_6_H_4_OH)_2_ is the BPA. The oxygen species O^–^ that is available for the reaction is formed due to electron transfer from the conduction band of Ag_2_O nanocubes to the adsorbed dissolved oxygen (in bulk solution or surface-air of an adjacent atmosphere). Detailed reactions are presented in eqs 2, 3, and 4
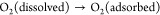
2
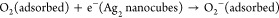
3
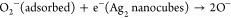
4

Using the Randles–Sevcik equation, it was found that 2.03 and 1.80 electrons are transferred in the anodic and cathodic halves of the redox reaction of BPA. This is approximately equal to 2, and therefore, a two-electron-transfer process is considered (as reported in our previous work^25^), which also matches with other literature^26,27^ where nanomaterial-based surfaces are used for specific detection of BPA with the nanomaterial being oxidized within the chosen range of potentials. More details of experiments including specificity and sensitivity of the redox detection of BPA are given in our previous work.^25^

In our analytical modeling, each of these scan rates (from 20 to 400 mV/s) was selected to study the movement of the redox reaction under different experimental conditions. First, we divide the data into two parts: one corresponding to the reduction phase and the other corresponding to the oxidation phase of the redox reaction. The measured total instantaneous current, *i*, during any of these phases is given by eq 5
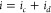
5where *i*, *i*_*c*_, and *i*_*d*_ are total, capacitive, and diffusive currents, respectively. This total current can also be expressed as eq 6
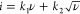
6where *i*_*c*_ = *k*_1_ν, *i*_*d*_ = *k*_2_*√ν*, and *k*_1_ is the capacitive current limiting constant and *k*_2_ is the diffusive-controlled current constant. Rearranging eq 6 generates eq 7.
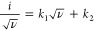
7

**Figure 2:**
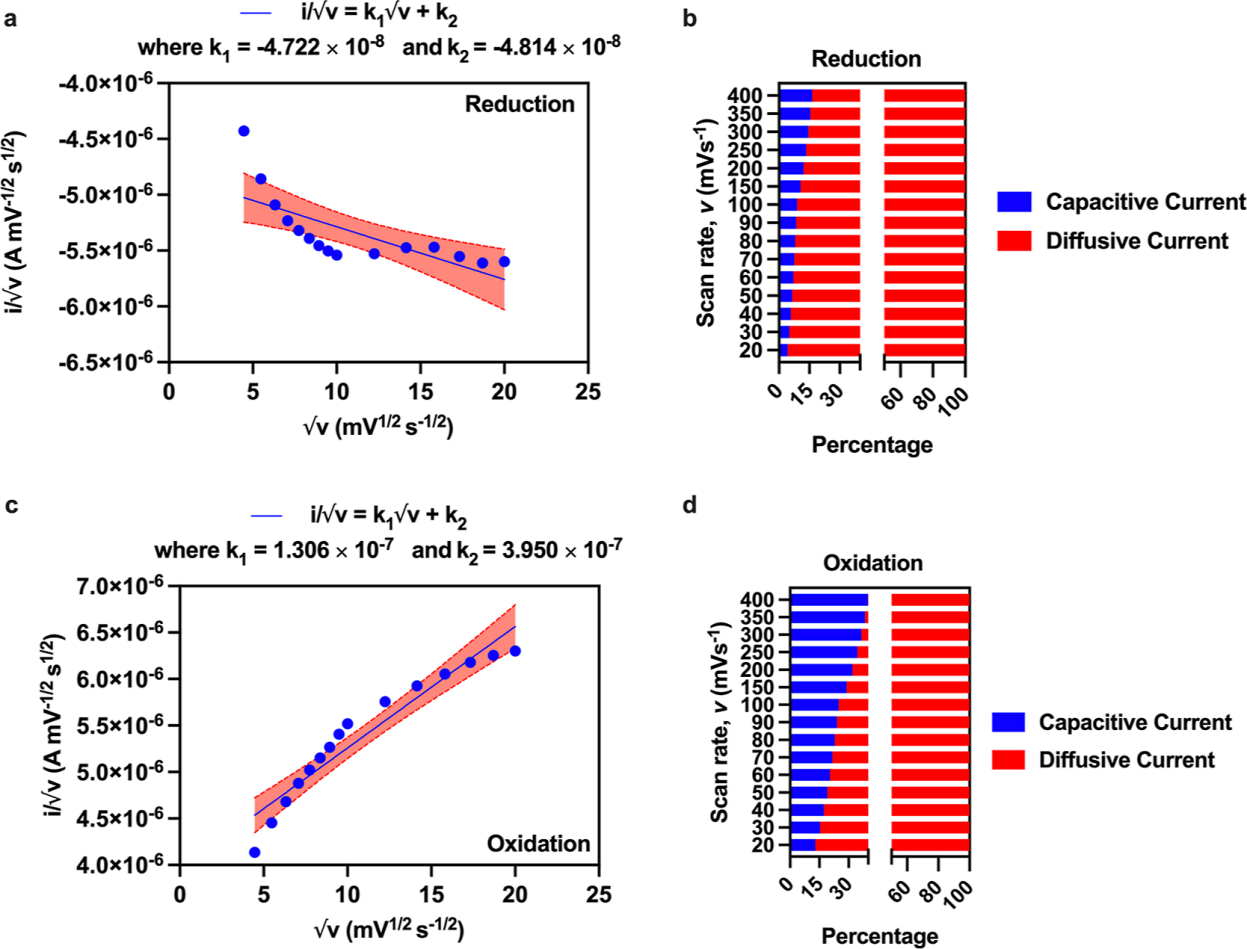
Capacitive and oxidative decoupling currents: (a) plot showing the reduction current divided by the square root of the scan rate versus the square root of the scan rate. (b) Percentage of capacitive and diffusive currents during the reduction phase. (c) Plot showing the oxidation current divided by the square root of the scan rate versus the square root of the scan rate and (d) percentage of capacitive and diffusive currents during the oxidation phase.

We first analyze the peak currents in the CV curves in Figure 1a, the corresponding peak potentials for oxidation and reduction phases shared in column 1 of Tables S1 and S2. The linear fitting achieved using eq 7 fits the reduction phase in Figure 2a and provides the values of *k*_1_ and *k*_2_. Using *k*_1_, *k*_2_, and ν, we find *i*, *i*_*c*_, and *i*_*d*_ and plot the percentage of capacitive limiting and diffusive components in the reduction phase in Figure 2b. Similarly, using eq 7, we fit the oxidation phase in Figure 2c and find the percentage of capacitive and diffusive currents in the reduction phase, Figure 2d. It should be noted that in our analysis, *k*_1_*v* indicates a double-layer capacitive contribution, whereas *k*_2_*v*^1/2^ is a semi-infinite diffusive faradaic contribution.^28^ It is interesting to note that the percentage of capacitive currents is higher during the oxidation phase compared with that during the reduction phase. This occurs because, during oxidation, the electrode initially charges up. In contrast, during reduction, the previously oxidized charge is discharged. Consequently, the charges stored in the capacitor formed during the oxidation phase are neutralized before any capacitance because the reduction reaction can build up on the electrode’s surface.

**Figure 3:**
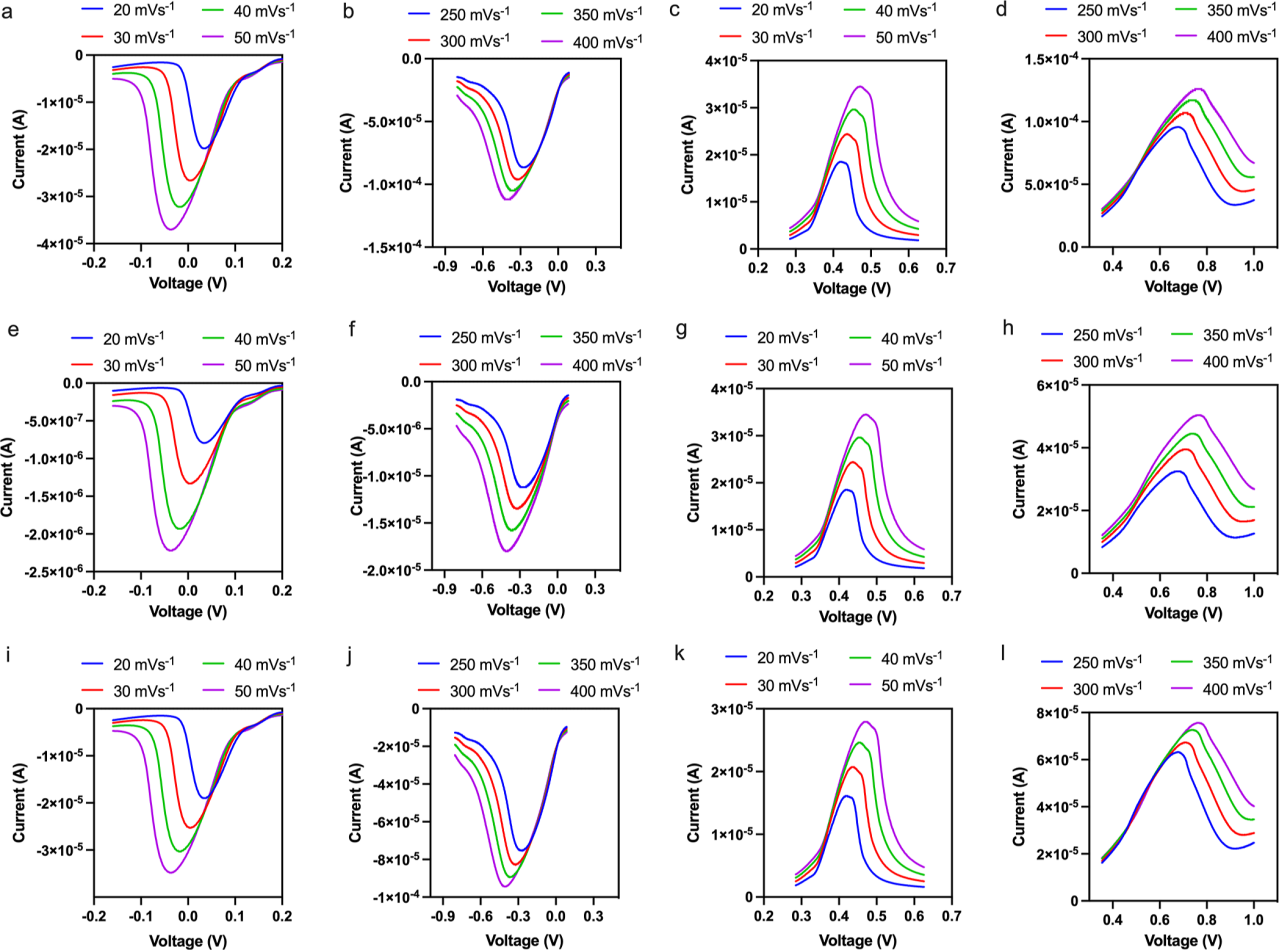
Oxidation and reduction phase voltammograms. (a,b) display reduction phase voltammograms with scan rates of 20–50 and 250–400 ms^–1^, respectively. (c,d) present oxidation phase voltammograms with the same respective scan rates. (e,f) show reduction phase voltammograms with scan rates of 20–50 and 250–400 ms^–1^, highlighting capacitive currents, while (g,h) depict oxidation phase voltammograms under the same conditions, also highlighting capacitive currents. (i,j) display reduction phase voltammograms with scan rates of 20–50 and 250–400 ms^–1^, showing diffusive currents. Finally, (k,l) present oxidation phase voltammograms with scan rates of 20–50 and 250–400 ms^–1^, respectively, also showing diffusive currents.

**Figure 4:**
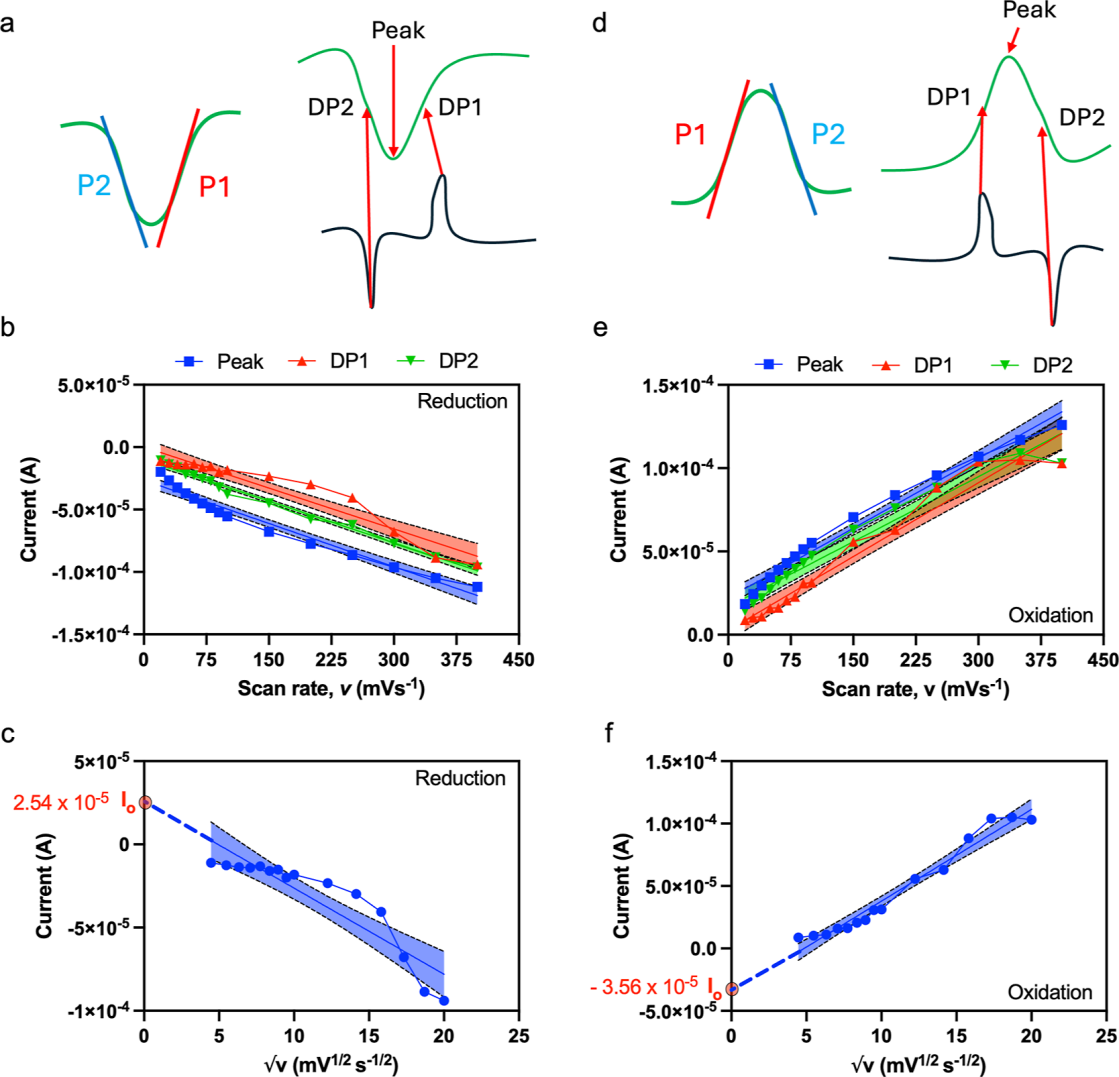
Flex point analysis. (a) displays the two slopes, P1 and P2, in the reduction phase of the measurement along with the corresponding DP1 and DP2 inflection points. The peak current is indicated and labeled as “Peak”. (b) illustrates the reduction current changes at the Peak, DP1, and DP2 points. (c) shows the current versus the square root of the scan rate for DP1 in the reduction phase. (d) displays the two slopes, P1 and P2, in the oxidation phase of the measurement along with the corresponding DP1 and DP2 inflection points. The peak current is indicated and labeled as “Peak”. (e) illustrates the oxidation current changes at the Peak, DP1, and DP2 points. (f) shows the current versus the square root of the scan rate for DP1 in the oxidation phase.

We then find the total current and its deconvoluted capacitive and diffusive currents, Figure 3a–i, based on the calculated percentages of capacitive and diffusive currents. Within this shape of current response, we have 2 arms (slopes) that we call P1 (leading arm) and P2 (trailing arm), see Figure 4a,d, for reduction and oxidation peaks, respectively. Now looking carefully at Figure 3, we can observe that the diffusive current mirrors the shape of the total current, while the arm P1 of the capacitive current shows greater voltage variations. Additionally, we know from literature that the peak voltage shifts due to the capacitive current.^18^ Based on this observation, we can also infer that the capacitive current plays a more significant role in voltage shifting. And in this context, it is P1 which shows more variations, and therefore, the capacitive properties can be studied using P1, while the diffusive properties can be studied using P2.

To quantify P1 and P2, we find the flex point, i.e., the current values where the first differential of the total current changes their curvature or essentially the point of maximum in the first differential. We call these points as DP1 and DP2, referring to differential point 1 and differential point 2, respectively. This provides us with 3 points, including the peak point, in each curve of oxidation and reduction curves. The current values at these 3 points are plotted for both reduction and oxidation including currents, Figure 4b,e, respectively. The current values for the DP1 point (point with maximum current change) are also plotted as a function of square root of the scan rate individually for the analysis of capacitive and diffusive currents in reduction, Figure 4c, and oxidation, Figure 4f, phases.

**Figure 5:**
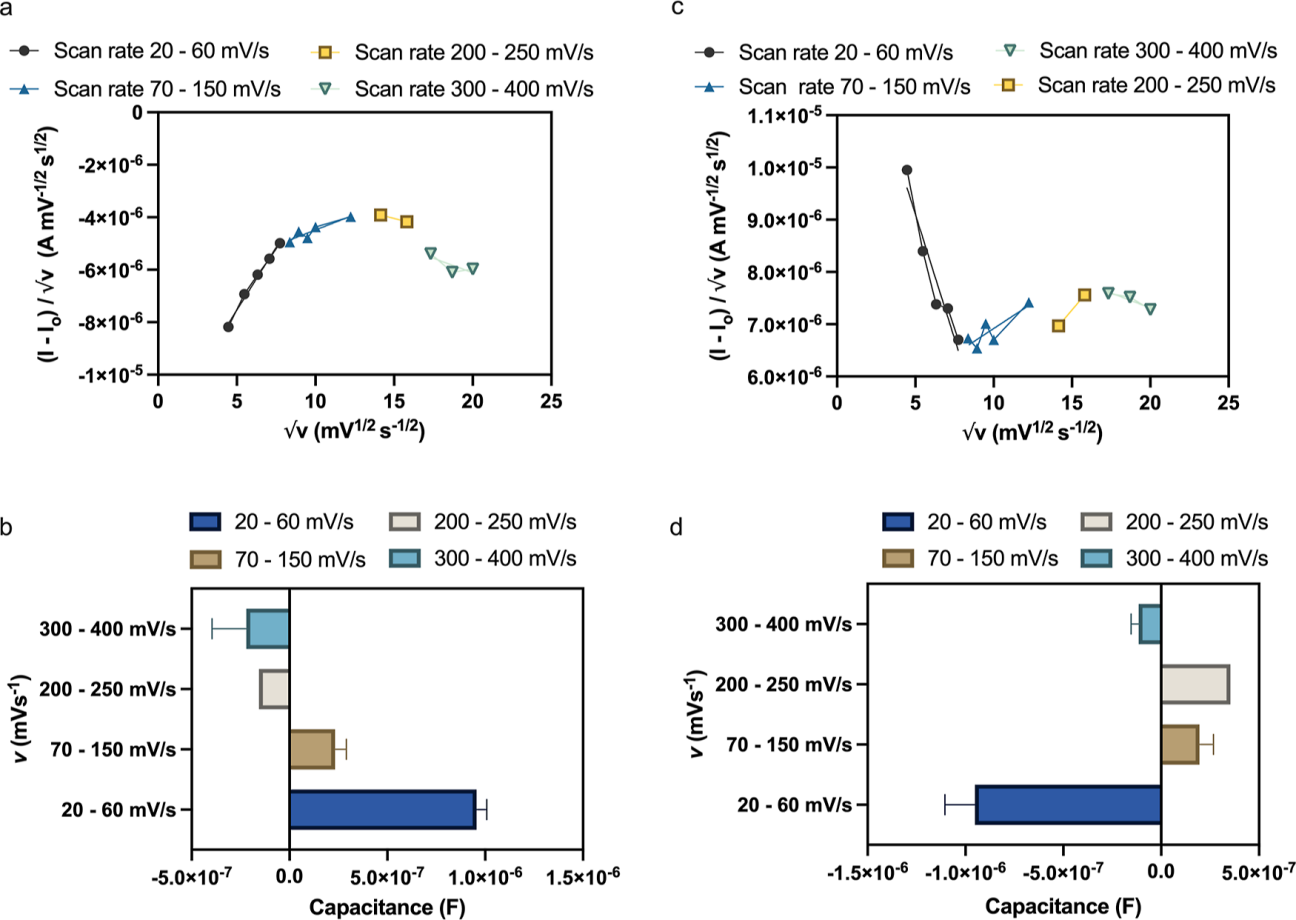
Capacitance analysis: (a) variation of (*I* – *I*_0_)/√ν with √ν for the reduction phase. (b) Variation of the capacitance in the reduction phase with the scan rate. (c) Variation of (*I* – *I*_0_)/√ν with √ν for the oxidation phase. (d) Variation of the capacitance in the oxidation phase with the scan rate.

These currents can be expressed by eq 8
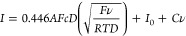
8which can be rewritten as eq 9
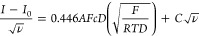
9In eqs 8 and 9, *A* is the area of the electrode, *F* is the Faraday constant, *c* is the bulk concentration of the species in the solution, *R* is the gas constant, and *D* is the diffusion constant. Additionally, *I* is the instantaneous current value at a given scan rate, and *I*_0_ is a current constant which is, empirically supported by experimental results, equal to the *y*-intercept of the linear fit (see Figure 4c,f). The slope of eq 9 will provide us with the capacitance, and therefore, we plot (*I* – *I*_0_)/√ν versus √ν and perform the piecewise linear fitting of this curve to find the variable capacitance. Please note that if all of the points can be fitted to a single linear line, the system will be considered to have a single capacitance value. Figure 5a shows the fitted slopes with different scan rates for the reduction phase, and the corresponding capacitance values are presented in Figure 5b. Similarly, the fitted slopes and corresponding variable capacitance values for oxidation phases are shown in Figure 5c,d, respectively. The value of capacitance varies between −0.5 and 0.5 μF which is the order of capacitance measured or expected for such systems (dielectric capacitance) reported in the literature.^29,30^ It is interesting to see the polarity change in the capacitances of both the reduction and oxidation phases at higher scan rates. Essentially, in the reduction phase, the capacitance is positive up to 150 mV/s scan rate compared to that in the oxidation phase where the negative capacitance starts to change its polarity to positive after 70 mV/s and then again starts to become negative after 300 mV/s.

This anomaly is attributed to the voltage and the charge state of the electrode where the rate of voltage change alters the types of ions (including their charge) that are favorable to accumulate on the surface.^31,32^ This, in turn, affects the structure and thickness of the electric double layer (EDL). Regions where specific ions are more readily adsorbed will result in a thicker EDL and consequently higher capacitance. Conversely, regions where less favorable ions accumulate will lead to a thinner EDL and lower capacitance. Additionally, at faster scan rates, the double layer may not have sufficient time to fully form and reach its equilibrium state at each applied voltage, leading to surface complexation.^3^ This results in lower capacitance, with variable charge, compared to slower scan rates where the EDL has more time to adapt to the changing potential.^33−35^ Furthermore, the variable capacitance could arise from the interplay of double-layer capacitance and charges generated by redox reactions.^36^ This is because the double-layer capacitance is caused by the orientation of dipoles aligning with the electric field from the electrode surface, while the charges generated by redox reactions orient in the opposite direction. Therefore, this variable capacitance observed in our analysis is a direct consequence of the dynamic changes in the EDL composition due to the interplay among the applied voltage, electrode material, and electrolyte solution. The scan rate further influences the observed capacitance by affecting the time allowed for EDL formation at each voltage point.

## Conclusions

Our research demonstrates that the capacitive and diffusive contributions of the redox currents can be effectively distinguished and deconvoluted. We provide a standardization method for analyzing minute variations in the leading and lagging slopes of the oxidation and reduction peaks. Additionally, our approach allows for the determination of the capacitance associated with the redox reaction. This work is useful for improving the accuracy of electrochemical measurements, which has broader implications in fields such as energy storage,^37^ sensor technology,^38^ and materials science.^39^ By enabling more precise characterization of redox processes, our method can contribute to the development of more efficient batteries, more sensitive detection methods, and advanced materials with tailored electrochemical properties.

## Methods

This study utilized raw CV data, specifically current versus voltage measurements. The data is used as such without any treatment from Figure 4a of our previous work.^25^ This was generated from an μ-Autolab PGSTAT (Metrohm) potentiostat attached to a computer having the general purpose electrochemical system software. The data was divided into two phases: reduction and oxidation. The peaks in each phase were identified to find the inflection points. The currents corresponding to these inflection points were further analyzed and compared as shown by our results. All data was processed using MATLAB and plotted using GraphPad Prism. Step-by-step details of the methodology are provided in the Supporting Information.

## Supporting Information Available

Detailed methodology, Matlab code for oxidation and reduction peak analysis, and potential values of peak and two inflection points for the oxidation and reduction phases of the reaction (PDF)
