# Supplementary material for: Decoupling Variable Capacitance and Diffusive Components of Active Solid–Liquid Interfaces with Flex Points
Source: ACS Meas Sci Au. 2024 Aug 29;4(5):599–605. doi: 10.1021/acsmeasuresciau.4c00057 (PMC11487932; doi:10.1021/acsmeasuresciau.4c00057)
Supplement: Supplementary file 1 — tg4c00057_si_001.pdf [file tg4c00057_si_001.pdf]

Supporting information

Decoupling variable capacitance and diffusive components of active solid-liquid interfaces with flex points

Liam Deehan,† Ajeet Kaushik ,‡ Ganga Ram Chaudhary,¶ Pagona Papakonstantinou,† and Nikhil Bhalla∗,†

†Nanotechnology and Integrated Bioengineering Centre (NIBEC) School of Engineering, Ulster University, 2-24 York Street, Belfast BT15 1AP, Northern Ireland, United Kingdom

‡Department of Environmental Engineering, Florida Polytechnic University, Lakeland, FL 33805, United States

¶Department of Chemistry and Centre of Advanced Studies in Chemistry, Panjab University, Chandigarh 160 014, India

Email: n.bhalla@ulster.ac.uk

Methodology

The unprocessed experimental data from the cyclic voltammetry experiments was documented in an excel file, where each scanning rate was assigned to its own sheet in the workbook. This organisation made it simple to access and handle the data for further analysis. Graphs were generated in excel to visually represent the cyclic voltammogram data. Each graph displayed the relationship between the applied potential (x-axis) and the current response (y-axis) for a particular scan rate. Graphical representation made it possible to qualitatively evaluate the redox behaviour, including the identification of oxidation and reduction peaks, see Figure S1.

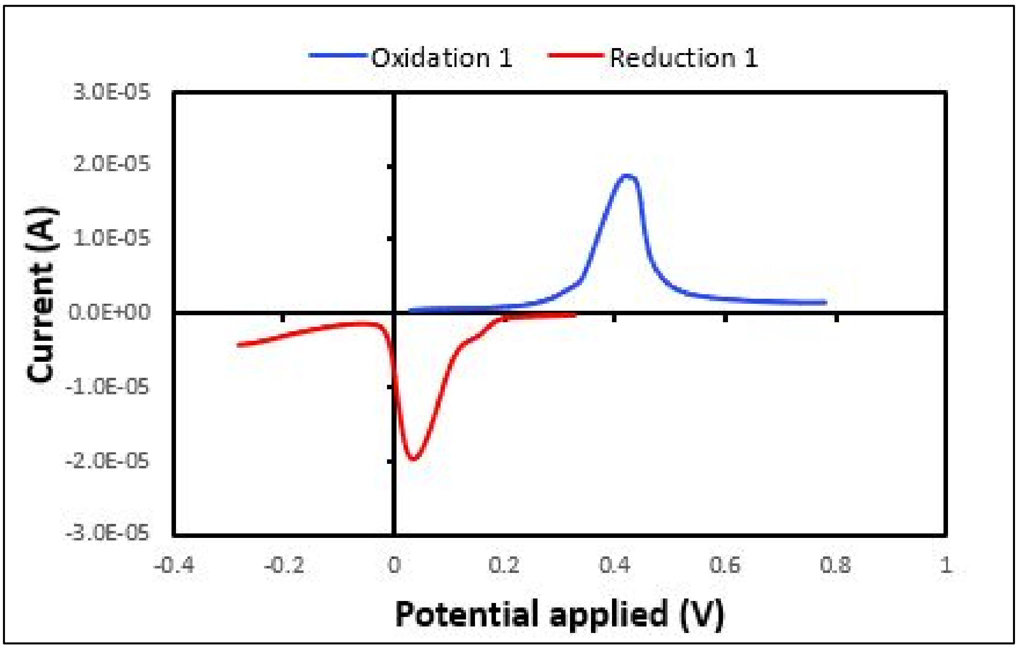

Figure S1. Isolated oxidation and reduction regions

Once the relevant data regions were identified and isolated, the data was formatted appropriately within excel. This involved organising the data into columns corresponding to the potential and current values for the oxidation and reduction curves. The formatted data was then exported from excel into individual MATLAB (.mat) files for further processing and analysis. The individual MATLAB files containing the formatted cyclic voltammetry data were loaded into MATLAB for subsequent analysis. MATLAB scripts were developed to perform data processing tasks, including differentiation, peak detection, and inflection point detection. Following code was developed:

Code for analysis of oxidation peaks

```
% Specify the directory containing the Results files
directory = 'C:\Users\......; % Update this to your directory

% Get a list of all .mat files in the directory
files = dir(fullfile(directory, '*.mat'));
fileNames = {files.name};

% Initialize the table to store results
results = table([], [], [], [], [], [], [], 'VariableNames', ...
            {'FileName', 'OriginalPeakX', 'OriginalPeakY', 'DerivativePeakX',
'DerivativePeakY', 'DerivativeTroughX', 'DerivativeTroughY'});

for fileIndex = 1:length(fileNames)
  fileName = fileNames{fileIndex}; % Use the variable from the loop for file name

  % Attempt to load the data and process
  try
    loadedData = load(fullfile(directory, fileName)); % Load the data
    var_name = who('-file', fullfile(directory, fileName));
    Res = loadedData.(var_name{1}); % Directly access the variable

    x = Res.Oxi_VarName1;
    y = Res.Oxi_MVs1;

    % Calculate the derivative of the data
    dy = diff(y) ./ diff(x);
    dx = (x(1:end-1) + x(2:end)) / 2; % Mid-points of x for derivative plot

    % Find peak and trough in the derivative data
    [maxDY, maxIdx] = max(dy);
    [minDY, minIdx] = min(dy);
    peakDX = dx(maxIdx);
    troughDX = dx(minIdx);

    % Find the closest original x values to the peakDX and troughDX
    [~, peakOriginalIdx] = min(abs(x - peakDX));
    [~, troughOriginalIdx] = min(abs(x - troughDX));
    peakX_original = x(peakOriginalIdx);
    troughX_original = x(troughOriginalIdx);
    peakY_original = y(peakOriginalIdx);
    troughY_original = y(troughOriginalIdx);

    % Find the maximum value in the original data and its corresponding x value
    [maxY, idx] = max(y);
    maxX = x(idx);

    % Append the results for this file to the results table
    newRow = {fileName, maxX, maxY, peakX_original, peakY_original,
troughX_original, troughY_original};
```

```
        results = [results; newRow];
    catch ME
        fprintf('%s: %s Skipping file.\n', fileName, ME.message);
    end
end

% Specify the filename for the Excel file to save the summary results
excelFileName = fullfile(directory, 'Oxidation Results 2.0.xlsx');

% Write the table to an Excel file
writetable(results, excelFileName);

% Display a message indicating completion
disp(['Results saved to ', excelFileName]);
```

Code for analysis of reduction peaks

```
% Specify the directory containing the Results files
directory = 'C:\Users\....'; % Update this to your directory

% Get all .mat files in the directory
files = dir(fullfile(directory, '*.mat'));
fileNames = {files.name};

% Initialize the table to store results
results = table([], [], [], [], [], [], [], 'VariableNames', ...
                {'FileName', 'OriginalTroughX', 'OriginalTroughY', 'DerivativePeakX',
'DerivativePeakY', 'DerivativeTroughX', 'DerivativeTroughY'});

for fileIndex = 1:length(fileNames)
    fileName = fileNames{fileIndex}; % Use the variable from the loop for file name

    % Attempt to load the data and process
    try
        loadedData = load(fullfile(directory, fileName)); % Load the data
        var_name = who('-file', fullfile(directory, fileName));
        Res = loadedData.(var_name{1}); % Directly access the variable

        x = Res.Red_VarName4;
        y = Res.Red_MVs2;

        % Calculate the derivative of the data
        dy = diff(y) ./ diff(x);
        dx = (x(1:end-1) + x(2:end)) / 2; % Mid-points of x for derivative plot

        % Find peak and trough in the derivative data
        [maxDY, maxIdx] = max(dy);
        [minDY, minIdx] = min(dy);
        peakDX = dx(maxIdx);
```

```
        troughDX = dx(minIdx);

        % Find the closest original x values to the peakDX and troughDX
        [~, peakOriginalIdx] = min(abs(x - peakDX));
        [~, troughOriginalIdx] = min(abs(x - troughDX));
        peakX_original = x(peakOriginalIdx);
        troughX_original = x(troughOriginalIdx);
        peakY_original = y(peakOriginalIdx);
        troughY_original = y(troughOriginalIdx);

        % Find the minimum value in the original data and its corresponding x value
        [minY, idx] = min(y);
        minX = x(idx);

        % Append the results for this file to the results table
        newRow = {fileName, minX, minY, peakX_original, peakY_original,
troughX_original, troughY_original};
        results = [results; newRow];
    catch ME
        fprintf('%s: %s Skipping file.\n', fileName, ME.message);
    end
end

% Specify the filename for the Excel file to save the summary results
excelFileName = fullfile(directory, 'Reduction Results 2.0.xlsx');

% Write the table to an Excel file
writetable(results, excelFileName);

% Display a message indicating completion
disp(['Results saved to ', excelFileName]);
```

The identified inflection points were compared with the corresponding points in the original cyclic voltammogram data. One example of oxidation and reduction is shared below in Figure S3 and Figure S4.

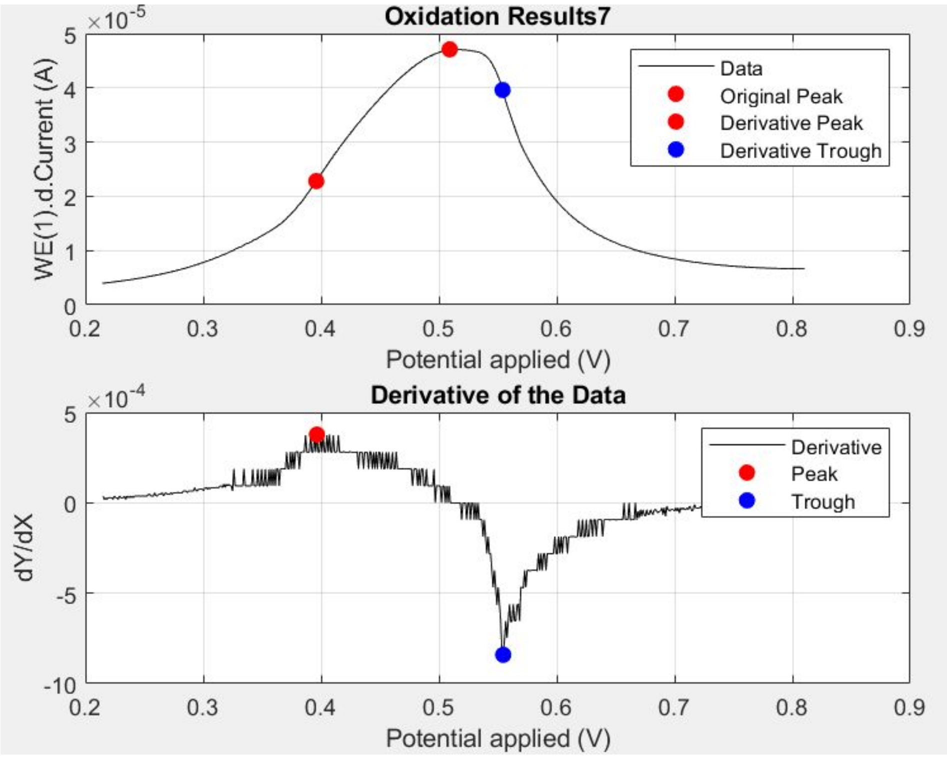

Figure S2. Oxidation peak analysis

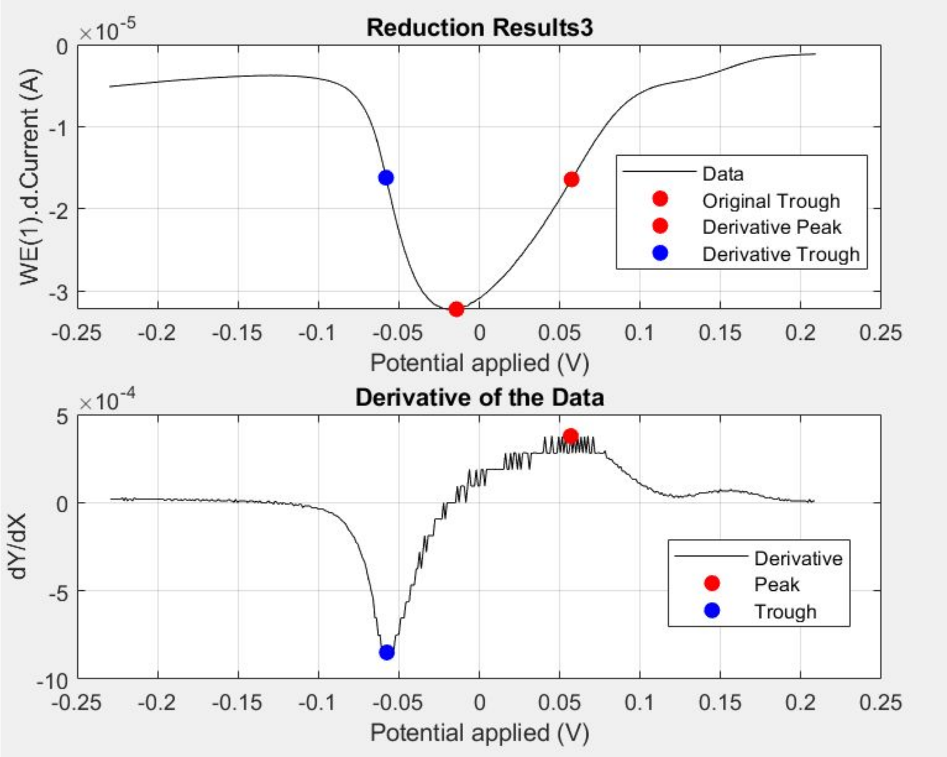

Figure S3. Reduction peak analysis

We also provide the peak potential values and DP1 and DP2 point for both reduction and oxidation phases.

Table S1. Potential values in oxidation phase

Scan Rate	Peak Potential	DP1 Potential	DP2 Potential
20	0.42053	0.361255	0.447765
30	0.43549	0.36232	0.4702
40	0.45044	0.36232	0.491565
50	0.46967	0.377275	0.50972
60	0.48248	0.378345	0.524675
70	0.4985	0.39116	0.539625
80	0.50919	0.3965	0.554585
90	0.52734	0.41893	0.568465
100	0.53909	0.41893	0.580215
150	0.59143	0.43068	0.633625
200	0.6395	0.51506	0.677415
250	0.67474	0.47554	0.715865
300	0.69931	0.678485	0.74257
350	0.72281	0.65712	0.78209
400	0.75058	0.62935	0.86113

Table S2. Potential values for reduction phase

Scan Rate	Peak Potential	DP1 Potential	DP2 Potential
20	0.03708	0.08461	0.003435
30	0.00717	0.07393	-0.030745
40	-0.01419	0.05684	-0.05745
50	-0.03449	0.06218	-0.078815
60	-0.05264	0.06218	-0.10231
70	-0.06439	0.05043	-0.121535
80	-0.08041	0.05043	-0.14183
90	-0.09323	0.028	-0.156785

100	-0.10925	0.03975	-0.17067
150	-0.15945	0.01732	-0.24971
200	-0.21606	-0.00618	-0.308455
250	-0.2652	-0.03929	-0.378955
300	-0.3186	-0.054245	-0.4142
350	-0.34424	-0.32875	-0.382155
400	-0.38055	-0.35759	-0.43663
